# Combined Catalytic Conversion of NOx and VOCs: Present Status and Prospects

**DOI:** 10.3390/ma18010039

**Published:** 2024-12-25

**Authors:** Mengzhao Li, Rui Wang

**Affiliations:** School of Environmental Science and Engineering, Shandong University, Qingdao 266237, China

**Keywords:** nitrogen oxide, volatile organic compound, catalysis, combined conversion

## Abstract

This article presents a comprehensive examination of the combined catalytic conversion technology for nitrogen oxides (NOx) and volatile organic compounds (VOCs), which are the primary factors contributing to the formation of photochemical smog, ozone, and PM2.5. These pollutants present a significant threat to air quality and human health. The article examines the reaction mechanism and interaction between photocatalytic technology and NH_3_-SCR catalytic oxidation technology, highlighting the limitations of the existing techniques, including catalyst deactivation, selectivity issues, regeneration methods, and the environmental impacts of catalysts. Furthermore, the article anticipates prospective avenues for research, underscoring the necessity for the development of bifunctional catalysts capable of concurrently transforming NOx and VOCs across a broad temperature spectrum. The review encompasses a multitude of integrated catalytic techniques, including selective catalytic reduction (SCR), photocatalytic oxidation, low-temperature plasma catalytic technology, and biological purification technology. The article highlights the necessity for further research into catalyst design principles, structure–activity relationships, and performance evaluations in real industrial environments. This research is required to develop more efficient, economical, and environmentally friendly waste gas treatment technologies. The article concludes by outlining the importance of collaborative management strategies for VOC and NOx emissions and the potential of combined catalytic conversion technology in achieving these goals.

## 1. Introduction

Against the backdrop of increasing global attention to environmental protection and the growing demand for reducing automobile exhaust emissions, the development of efficient and stable catalysts and post-treatment systems has become particularly important [1]. NOx and VOCs are two common types of pollutants in industrial emissions. They not only pose a threat to human health, such as causing respiratory diseases and increasing the risk of cancer [2], but also have serious environmental impacts, including the formation of photochemical smog, acid rain [3], and damage to the ozone layer. In addition, NOx and VOCs are key precursors for the formation of ground level ozone and fine particulate matter (PM2.5), which often exceed environmental standards in many areas and pose a serious threat to air quality and human health [4,5]. Taking Qinhuangdao City as an example, the main atmospheric pollutant emissions in 2016 were as follows: the annual emissions of nitrogen oxides (NOx) were 86.83 tons, the annual emissions of volatile organic compounds (VOCs) were 52.69 tons, and the annual emissions of particulate matter (PM10 and PM2.5) were 302.01 tons and 116.85 tons, respectively [6,7]. In VOCs emissions, industrial processes are the main source, accounting for 40.55%, with the steel industry making the most significant contribution, accounting for approximately 26.4% of total VOCs emissions [6,8,9]. In addition, fossil fuel combustion and mobile sources account for 13.5% and 12.9% of the total VOCs emissions, respectively. The main sources of NOx emissions are ships and agricultural transport vehicles. As for particulate matter (PM), its main emission sources include industrial processes and dust sources [10,11,12,13]. The flue gas of important pillar industries such as steel, coal, and coking usually contains volatile organic compounds (VOCs) and nitrogen oxides (NOx) [14,15,16]. At present, the main methods for treating these pollutants include adsorption technology, which is a widely researched and applied concentration technology [17,18,19]. Although adsorption technology can effectively enrich pollutants, it has not achieved thorough treatment of pollutants [20]. In addition, currently efficient adsorbents often have disadvantages such as high toxicity, high cost, and complex preparation processes, which limit their widespread application in industrial production [21,22,23,24]. Another method is degradation technology, which includes direct combustion [25,26,27] and catalytic methods [28,29,30]. Among these methods, catalytic technology stands out due to its ability to efficiently convert pollutants into harmless substances [31,32]. This technology has low energy consumption during processing and does not require complex equipment [33,34]. Due to the similar optimal treatment temperatures for NOx and VOCs, it is possible to use a single device to simultaneously treat these two pollutants, which not only improves efficiency but also brings significant social and economic benefits [4,35,36]. This is particularly important for industries with limited space for technological upgrades. Therefore, there is an urgent need to implement a collaborative management strategy for VOCs and NOx emissions.

Recently, there has been a breakthrough in the research field of synergistic catalytic conversion of NOx and VOCs. Researchers are actively developing a class of bifunctional catalysts that can simultaneously complete the reduction reaction of NOx and the oxidation reaction of VOCs over a wide temperature range [37,38,39]. The design of catalysts usually involves adjusting the acid-base properties and redox ability of the catalyst, as well as delving into its reaction mechanism and the interactions between different components. By using methods such as metal doping [40], surface modification [41], selection of suitable carrier materials [42], and control of catalyst morphology [43], researchers have optimized the performance of catalysts and enhanced their stability and durability in industrial applications [44]. In addition, the study of the poisoning mechanism and regeneration process of catalysts in complex gas environments provides a theoretical basis for ensuring the long-term stable operation of catalysts [45,46,47].

In the current research field, the combined catalytic technologies for VOCs and NOx mainly include selective catalytic reduction (SCR) technology [38,48,49,50,51,52,53,54], photocatalytic oxidation technology [55,56], low-temperature plasma catalytic technology [57,58,59], and biological purification technology [60,61]. Among them, the method of simultaneously eliminating NOx and VOCs by implementing SCR and catalytic oxidation reactions on bifunctional catalysts has become a focus of research in the field. These catalysts are capable of simultaneously completing the reduction reaction of NOx and the oxidation reaction of VOCs across a wide temperature range. The design of catalysts typically entails the manipulation of the catalyst’s acid–base properties and redox ability, as well as a comprehensive investigation of its reaction mechanism and the interactions between its various components. Researchers have optimized the performance of catalysts and enhanced their stability and durability in industrial applications through techniques including metal doping, surface modification, the selection of appropriate carrier materials and the control of catalyst morphology. Furthermore, the investigation of the poisoning mechanism and regeneration process of catalysts in complex gas environments provides a theoretical foundation for ensuring the long-term stable operation of catalysts.

Despite the numerous breakthroughs that have been made in laboratory research, there are still a number of challenges that need to be overcome before these achievements can be applied to practical industrial production. This encompasses the durability of catalysts under authentic industrial circumstances, the synchronous efficacy of the treatment for multiple pollutants, and the adaptability of catalysts to diverse operational conditions. It is therefore recommended that future research should focus on a deeper understanding of the design principles of composite component catalysts, the structure–activity relationship of catalysts, and the performance evaluation of catalysts in real industrial environments. These studies will provide a robust scientific foundation for the development of more efficient, economical, and environmentally friendly industrial waste gas treatment technologies. This article presents a comprehensive review of recent research trends in the field of combined catalytic conversion of NOx and VOCs at home and abroad, and offers insights into future research directions.

## 2. Combination Catalytic Conversion Technology

Currently, the technology of the combined catalytic conversion of nitrogen oxide (NOx) and volatile organic compounds (VOCs) is gradually becoming a research frontier in the field of environmental pollution control. This technology aims to simultaneously remove these two pollutants through a single catalytic system in order to achieve more efficient pollution control effects. The main methods include the use of selective catalytic reduction (SCR) technology, photocatalysis technology, low-temperature plasma technology, biological purification technology, and the development of new combined catalytic oxidation catalysts. These technologies achieve the conversion of NOx and VOCs through different mechanisms, such as redox reactions, photochemical reactions, plasma excited-state reactions, and biological metabolic processes.

NH_3_-SCR technology, usually using ammonia as a reducing agent, converts NOx into nitrogen and water under the action of a catalyst, and is now also being studied for the oxidation of VOCs [48]. Photocatalytic technology utilizes light energy to excite catalysts and generate oxidative free radicals, which oxidize and decompose pollutants [55]. However, this technology is limited by the light conditions and requires an improved catalyst efficiency. Low-temperature plasma technology can effectively remove various pollutants, but there are issues with energy consumption and cost [27]. Biological purification technology is a low-cost solution, but its processing efficiency and speed are limited by environmental factors [60]. The following sections discuss the combined catalytic conversion technology.

### 2.1. NH_3_-SCR Catalytic Technology

NH_3_-SCR technology is a mature industrial exhaust treatment technology mainly used for removing NOx. Researchers are exploring its application in VOC catalytic degradation to achieve the synergistic removal of NOx and VOCs. SCR technology has the following advantages: synergistic removal of NOx and VOCs, high catalytic efficiency, commercially mature technology, adjustability, and environmental friendliness. However, it also faces challenges such as high catalyst costs, catalyst poisoning, limited reaction conditions, by-product issues, low selectivity for VOC oxidation, increased system complexity, and limited technological adaptability. Future research directions may include developing more economically stable catalysts and optimizing the reaction conditions to improve system performance and reliability.

#### 2.1.1. Vanadium (V)-Based Catalyst

**Figure 1:**
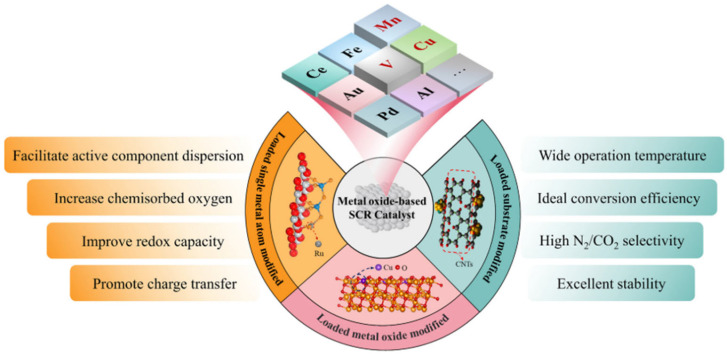
Different modification methods and the enhancement of their properties in different metal oxide-based SCR catalysts [36]. Copyright 2023, Elsevier.

Vanadium-based catalysts are some of the main catalysts used in selective catalytic reduction (SCR) technology for the removal of nitrogen oxide (NOx), and are widely used in coal-fired power plants and diesel vehicle exhaust treatment. Vanadium-based catalysts typically consist of vanadium oxide (V_2_O_5_) as the active component, which is loaded onto carriers such as TiO_2_ and WO_3_/TiO_2_ [62]. These catalysts exhibit efficient NOx reduction activity at moderate temperatures (350–400 °C). However, vanadium-based catalysts also have some limitations, such as insufficient activity at low temperatures, poor thermal stability, susceptibility to poisoning by substances such as SO_2_, and ammonia escape problems [40], as shown in Figure 1.

To overcome these limitations, researchers have conducted extensive modification studies to improve the performance of vanadium-based catalysts. The modification methods include the use of a second metal addition, carrier substitution, structural optimization, pretreatment, and post-treatment. For example, by adding transition metals such as Cu [63], Fe [64], Ce [65], Mn [38], Pd [41,66], Pt [67] and Ru [68] as cocatalysts, the surface acidity can be enhanced, NH_3_ adsorption capacity can be increased, and surface defects and oxygen vacancies can be increased, thereby providing more active sites. Of these, Pd and Pt have been successfully commercialized and are widely used in industrial applications for the combined catalytic conversion of NOx and VOCs. Fu et al. [69] designed a novel bilayer catalyst that combines two different functional layers, CoCeOx and V_2_O_5_/TiO_2_, for the simultaneous removal of nitrogen oxide (NOx) and volatile organic compounds (VOCs). Research has found that this double-layer catalyst exhibits a high NOx removal efficiency and good propane degradation performance over a wide temperature window. The conversion rate of NO can reach 100% at 225 °C and remains above 80% in the range of 175–300 °C, while the degradation of VOCs can reach 80% above 260 °C. The study also explored bilayer catalysts with different structures, revealing the mechanisms involved in this particular structure. The V_2_O_5_/TiO_2_ layer has abundant acid sites that can adsorb NH_3_ as an ammonia buffer, while the CoCeOx layer provides excellent oxidation ability to oxidize NO and propane. This combination regulates the competitive adsorption between NH_3_ and propane, which is the initial obstacle to the removal of multiple pollutants. The relevant vanadium-based catalysts are shown in Table 1.

**Figure 2:**
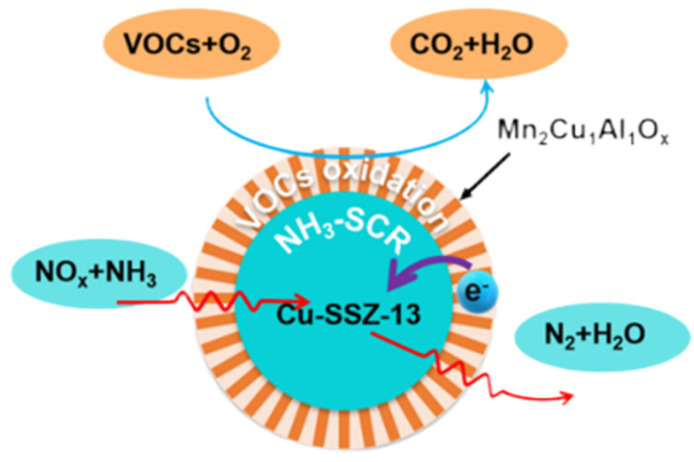
Schematic diagram of the mechanism for the simultaneous removal of toluene and NOx by the Cu-SSZ-13@Mn_2_Cu_1_Al_1_Ox catalyst [54]. Copyright 2016, Elsevier.

Wang et al. [75] studied the simultaneous reduction of methanol and NOx using a copper-modified Sb-Ce-Zr catalyst. The addition of copper has been demonstrated to enhance the oxidation of CH_3_OH and to alleviate its inhibitory effect on selective catalytic reduction (SCR). Furthermore, the addition of copper has been shown to improve the activation of SCR reactants in the presence of methanol. Nevertheless, the incorporation of elevated quantities of copper may disrupt the structural configuration of the Sb-Ce-Zr oxide solid solution, thereby reducing the specific surface area and acidity. The excessive addition of copper may also result in a narrowing of the temperature window for NOx conversion. These findings can provide valuable insights for the synergistic removal of VOCs by SCR catalysts in practical applications. Zhang et al. [54] designed a bifunctional compound with excellent activity using a Cu-SSZ-13@Mn_2_Cu_1_Al_1_Ox core-shell catalyst, as shown in Figure 2, for the simultaneous removal of VOCs and NOx. A core-shell bifunctional catalyst with multiple active sites was synthesized by the in situ growth of vertically oriented Mn_2_Cu_1_Al_1_Ox nanosheets on the surface of Cu-SSZ-13. The catalyst has been optimized and exhibits a 100% conversion rate for the simultaneous removal of volatile organic compounds (VOCs) and nitrogen oxide (NOx) at 300 °C and in the presence of 5% water vapor. The results of the physical and chemical characterization, in conjunction with the findings of the density functional theory (DFT) calculations, indicate that Cu-SSZ-13@Mn_2_Cu_1_Al_1_Ox exhibits a greater degree of surface acidity and an increased number of oxygen vacancies. The charge transfer between the core and shell is the intrinsic reason for the improvement in the removal activity of VOCs and NOx. The molecular orbital theory is used to explain the different adsorption energies caused by the different bonding modes between the core-shell and mixed individual catalysts. This work provides a new strategy for the design of efficient catalysts for the simultaneous removal of VOCs and NOx or other multiple pollutants.

#### 2.1.2. Manganese (Mn)-Based Catalyst

In NH_3_-SCR technology, Mn-based oxide catalysts have attracted widespread attention due to their low cost, environmental friendliness, ease of preparation, and high activity in VOC and NOx catalytic oxidation. Mn-based oxide catalysts include metal-doped MnOx, for example, by doping Ag [76], Au [77], Pd [78], Pt [78] and other metals to enhance the mobility and activity of surface oxygen species; the composite MnOx produces synergistic effects by combining with metals such as Fe [79], Ce [80], Cu, Co [81], V [82], Ti [83], Sn [84], and Ln [85]; and loaded Mn-based oxides, which are loaded on carriers such as Al_2_O_3_ [86], porous calcium silicate [87], HZSM-5 zeolite [88], etc., as shown in Table 2.

**Figure 3:**
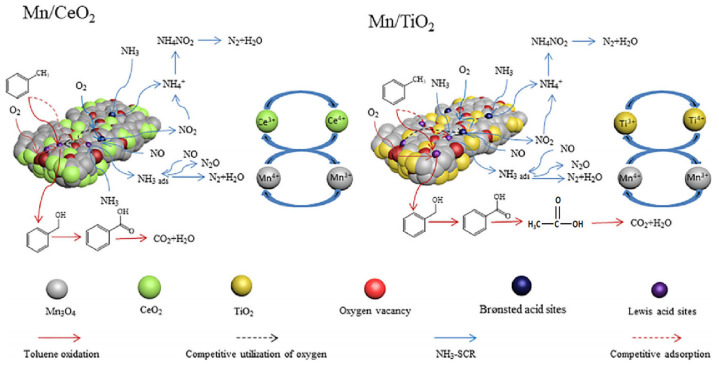
The mechanism of the simultaneous removal of toluene and NOx by Mn/CeO_2_ and Mn/TiO_2_ catalysts [89]. Copyright 2022, Elsevier.

For example, Pan et al. [89] used Mn/CeO_2_ and Mn/TiO_2_ catalysts to simultaneously remove NOx and toluene, with a removal rate of up to 80%. And it was found that there was mutual inhibition during the simultaneous removal process, mainly due to the occupation of Lewis acid sites by toluene adsorption, which inhibited the adsorption of NH_3_. At the same time, the reaction between NH_3_/NO and reactive oxygen species was more likely to occur, thereby inhibiting the oxidation of toluene. The mutual inhibition mechanism is shown in Figure 3.

In addition, special structures of Mn-based oxides, such as hollow microspheres, porous structures, and core-shell structures, were also mentioned, as well as Mn-based composites prepared by metal organic framework (MOF) derivation and a self-sacrificial template mode. These catalysts have shown potential in improving the efficiency of VOC catalytic oxidation, but their stability and activity still need to be further improved to address issues such as deactivation and promote their use in industrial applications.

#### 2.1.3. Precious Metal Catalysts

Among the numerous catalytic technologies, precious metal catalysts have received widespread attention due to their high activity, good stability, and easy regenerability. Precious metal catalysts mainly include platinum (Pt) [96], palladium (Pd) [97], gold (Au) [98], silver (Ag) [99], iridium (Ir) [100], etc. These metals are widely used for catalyzing the oxidation of VOCs due to their unique electronic structures and catalytic performance. Pt and Pd have become the most extensively studied catalysts due to their high activity and good stability at low temperatures. Au exhibits a distinctive performance in the catalytic oxidation of volatile organic compounds (VOCs). Despite its lower catalytic activity in comparison to Pt and Pd, its high dispersibility on oxide supports can enhance the catalytic oxidation of VOCs, and it has a higher CO_2_ selectivity than other precious metal catalysts. The relatively low price and synergistic effect with metal oxide carriers have attracted attention to Ag. It has been demonstrated that Ag can enhance the formation of active oxygen species in the catalytic oxidation process of VOCs, thereby improving the activity of catalysts. While the reaction rate of iridium (Ir)-based catalysts is typically lower than that of Pt- and Pd-based catalysts, they exhibit high sintering resistance in oxygen-containing atmospheres.

The methodology employed in the preparation of catalysts exerts a considerable influence on their activity, selectivity and stability. The most commonly utilized preparation techniques encompass the impregnation method, coprecipitation method, sol-gel method, and so forth. The implementation of disparate preparation techniques may engender alterations in the physical and chemical attributes of catalysts, which in turn may impact the catalytic performance of the catalysts in question. For example, Au/CoOx catalysts prepared by the coprecipitation method exhibit high efficiency in the complete combustion of propane [101], while Pt/Fe_2_O_3_ catalysts prepared by the impregnation method exhibit high activity in the catalytic oxidation of HCHO at low temperatures [102]. In addition, the pretreatment conditions of the catalyst, such as the calcination temperature and atmosphere, can also have significant impacts on its performance.

**Figure 4:**
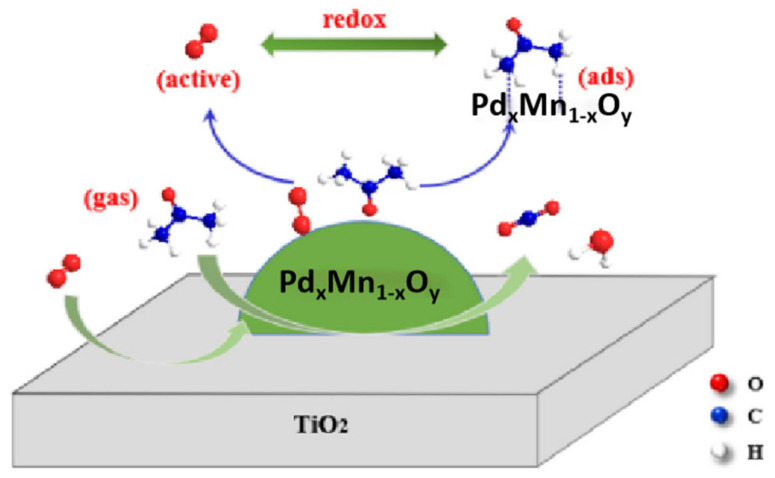
Proposed MVK mechanism over the PdMn/Ti catalyst [103]. Copyright 2019, Elsevier.

The addition of additives can significantly improve the performance of precious metal catalysts. Auxiliaries can produce synergistic effects with active components, alter the interactions between active components and carriers, and regulate the acidity of carriers. As shown in Figure 4, the addition of Mn can enhance the acetone oxidation activity of a Pd/TiO_2_ catalyst. Under the action of the PdMn/Ti catalyst, acetone in the gas phase is first adsorbed on the Pd_x_Mn_1−x_O_y_ active site of the catalyst, and then the adsorbed acetone migrates to the internal surface of the catalyst. At the same time, oxygen in the gas phase enters the oxygen vacancies on the catalyst surface and is activated to generate reactive oxygen species. Finally, the adsorbed acetone reacts with reactive oxygen species to produce carbon dioxide and water. This process involves the adsorption and activation of active sites on the catalyst surface, as well as the progress of redox reactions, reflecting key steps in the MVK mechanism [103], while the addition of Ce can provide more strong acid sites for Pd/Al_2_O_3_ catalyst [104]. The addition of Mo can enhance the dispersibility of Pd in Pd/Al_2_O_3_ catalysts, change the oxidation state of Pd species, and promote the adsorption of oxygen on the catalyst surface [105].

The choice of carrier is crucial for the performance of precious metal catalysts. Different carriers can affect the activity of catalysts due to differences in their physical and chemical properties. For example, CeO_2_ has emerged as a pivotal catalyst in heterogeneous catalytic oxidation, largely due to its plentiful oxygen vacancies, robust interactions with metals, and adaptable exposed crystal planes [99]. In addition, molecular sieves with a large specific surface area and a distinctive pore structure are regarded as superior carriers for the loading of active components. The acidity and alkalinity of the carrier are also significant factors influencing the activity of the catalyst. The appropriate acidity and alkalinity can enhance the activity and stability of the catalyst [106].

The study of the reaction mechanism of volatile organic compound (VOC) catalytic oxidation is of great importance for the design of high-performance catalysts. While it is challenging to propose a universal and unique reaction mechanism due to the varying properties of VOCs under different reaction conditions, researchers have conducted comprehensive research on intermediates and active species in the reaction process through experimental and theoretical calculation methods. For example, the catalytic oxidation of benzene on Pd-based catalysts is mainly carried out through a dehydrogenation oxidation process. Conversely, the oxidation of HCHO on a Pt/Co_3_O_4_-NiO catalyst is initially transformed into the intermediate dioxomethyl (DOM), which is subsequently decomposed into CO_2_ and H_2_O [107].

In the catalytic oxidation of volatile organic compounds (VOCs), the antisintering and anticarbon deposition properties of the catalyst are of paramount importance for ensuring its long-term stability. The activity of Ir-based catalysts can be effectively enhanced by the selection of appropriate carriers and the adjustment of the structure and chemical state of the catalyst. For instance, low-temperature calcination (350 °C) has been demonstrated to promote the formation of small Ir particles (approximately 5 nm), which in turn increases the proportion of defect Ir^3+^ species and highly reducible iridium species, thereby enhancing the activity of the catalyst [108].

The performance of these catalysts is influenced by a number of factors, including the size, dispersion, valence state, and metal support interaction of precious metal particles. Consequently, a significant proportion of studies concentrate on the characterization of carriers or metals, the methodology employed in the preparation of catalysts, and the impact of catalyst additives on the catalytic performance of VOC removal processes. Given the elevated cost of precious metal catalysts, it is also crucial to reduce the loading of precious metals and prepare catalysts with satisfactory activity and stability. Furthermore, it is essential to enhance the stability and service life of precious metal catalysts.

### 2.2. Low-Temperature Plasma Catalytic Technology

**Figure 5:**
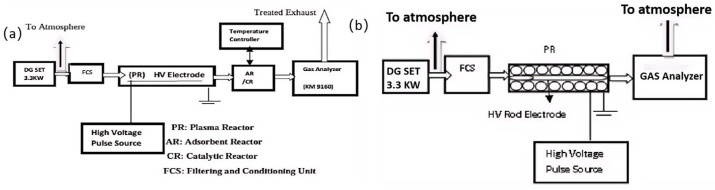
Diesel engine exhaust treatment using a single-step plasma augmented catalysis/adsorbent technique (**a**) and diesel engine exhaust treatment using a single-step packed bed reactor technique (**b**) [111]. Copyright 2019, Springer Nature.

Plasma technology is an advanced method that activates gas molecules by generating high-voltage discharges in the gas, thereby removing nitrogen oxide (NOx) and volatile organic compounds (VOCs) from exhaust gas [109]. Dielectric barrier discharge (DBD), a common method for generating non thermal plasma, generates a large amount of active free radicals and ions by forming a discharge between electrodes, promoting the reaction with pollutants in the exhaust gas and achieving harmless treatment [110]. By combining catalysts such as metals or metal oxides, the reaction efficiency and selectivity can be further improved. Pulse electrodynamic dielectric barrier discharge plasma hybrid technology (PDBDH) optimizes the removal efficiency of NOx and VOCs by adjusting pulse parameters [111]. Jagadisha N et al. [111] used PDBDH to simultaneously treat NOx, VOC, and CO emissions from fixed diesel engines. As shown in Figure 5, the experimental setup includes a high-voltage pulse power supply, diesel engine setup, plasma reactor, adsorbent and catalytic reactor, filtration and regulation unit, and measurement equipment. Different sizes of reactors, rod-shaped and linear electrodes, as well as filtered and unfiltered exhaust gases were used. Two plasma mixing configurations were studied: in the first configuration, the reactor was filled with activated alumina, alumina coated with silver nitrate, and red mud balls; in the second configuration, VOCs were first treated with plasma and then treated in series with red mud. Research has found that when using a plasma reactor alone, rod-shaped electrodes have a higher removal efficiency for all flow rates. The packed bed reactor filled with activated alumina coated with silver nitrate showed a removal performance of up to 95% for NOx. The removal rate of VOCs by the series plasma configuration was about 80%.

In addition, Li et al. [112] utilized a synergistic effect combining in plasma catalysis (IPC) and sodium sulfite (Na_2_SO_3_) wet scrubbing technology to simultaneously eliminate NOx and VOCs from a simulated sintering flue gas, with an optimal discharge power range of 10 to 17 watts and an NO conversion rate of 95.7%. In addition, the removal rate of VOCs can reach 88%. However, plasma technology still faces challenges in industrial applications, such as high initial equipment costs, maintenance requirements, by-product handling issues, challenges in scaling up to industrial scale, potential corrosion issues, ozone generation, and the complexity of technical operations.

### 2.3. Biological Purification Technology

Currently, there is a substantial body of research exploring the removal of volatile organic compounds (VOCs) and nitrous oxide (NO) through biological purification technology. Among these studies, chemical absorption with biological reduction (CABR) and biological drip filtration (BTF) have demonstrated considerable potential for the treatment of NO and VOCs, respectively [113,114]. The CABR method entails the removal of NOx through a two-stage process: firstly, chemical adsorption, and secondly, biological reduction. In the stage of chemical adsorption, NOx is absorbed by chemical chelating absorbents (such as Fe(II)-EDTA) to form complexes. This process mainly occurs in plate towers, simulating the complexation and absorption of NO by the flue gas through gas–liquid contact. The absorbed NOx complex then enters the bioreactor and is reduced through the action of microorganisms [60]. During this process, NOx is reduced by microorganisms to harmless nitrogen gas (N_2_). This step involves denitrification by microorganisms, reducing NO to N_2_ while thoroughly degrading organic matter in the liquid phase [115]. In general, CABR technology is more appropriate for NO enhancement treatments involving low temperatures, high humidity, a high air volume, and complex compositions. Conversely, SCR and SNCR methods are not applicable under such circumstances. However, chelating absorbents are prone to loss during the iron deposition process.

In the CABR system, the removal of VOCs is mainly achieved through a biotrickling filter (BTF) [115]. VOCs are degraded by microorganisms in packed towers and converted into alcohol ketone organic compounds and CO_2_. In the CABR system, multiple microorganisms were found to play a synergistic role in the system. For example, some microorganisms generate the electron donor H_2_ and consume dissolved oxygen while degrading VOCs, promoting bioreduction reactions and reducing the loss of chelating absorbents. Some microorganisms can reduce sulfites to S^2−^, causing some chelating absorbents to be lost in the form of ferrous sulfide (FeS) and undergo redox reactions with trivalent iron (Fe (III)) before entering the circulation system again.

**Figure 6:**
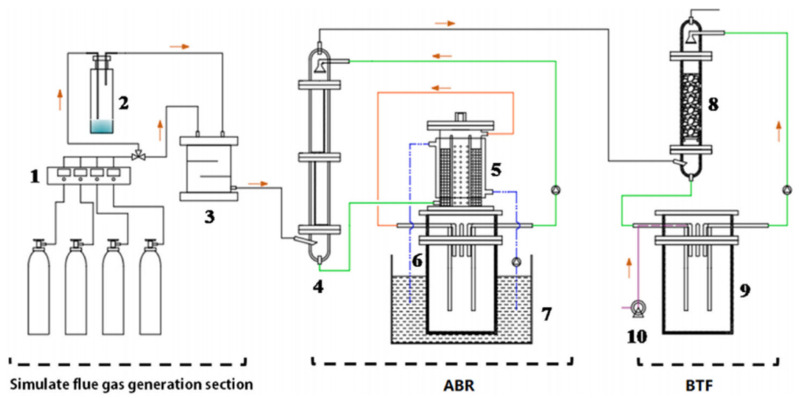
Schematic diagram of a joint system device. 1. Mass flow controller, 2. VOC vaporization chamber, 3. gas mixing chamber, 4. plate tower, 5. bioreactor, 6. storage tank, 7. water bath, 8. biological packing tower, 9. storage tank, 10. air pump. [61]. Copyright 2022, Elsevier.

Tong Wei et al. [61] constructed a coupled biotrickling filter (ABR-BTF) device for simultaneously removing nitrous oxide (NO) and volatile organic compounds (VOCs) from the flue gas of a WtE factory. Figure 6 illustrates the configuration of the novel ABR-BTF joint operation device, which comprises three principal sections: a simulated flue gas generation section, an absorption bioreactor (ABR) based on the CABR method, and a biological droplet filter (BTF). In this device, the chemical absorption bioreactor (ABR) is employed for the reduction of NO, while the biotrickling filter (BTF) is utilized for the elimination of toluene. Following a 30 s simulation of the flue gas passing through the system, the removal efficiencies of NO and toluene were found to be 85% and 90%, respectively. In comparison with a standalone ABR, the loss rate of NO composite absorbent in the ABR-BTF system was reduced by 85%. Concurrently, research has identified a multitude of novel bacterial genera within integrated systems, exhibiting promising capabilities in the domains of biological denitrification and volatile organic compound (VOC) elimination. Among these, the hydrogen gas produced by Candidatus_Cloacamonas and Syntrophomonas can function as electron donors, which are utilized by dissolved oxygen species during the VOC degradation process, thereby facilitating bioreduction and reducing the loss rate of ABR composite absorbents. Meanwhile, Tisirella and Desulfitobacterium are capable of reducing sulfites to S^2 −^, allowing some composite absorbents to be converted to FeS through redox reactions with Fe^3+^ and reintroduced into the circulation system. This integrated biological purification system is capable of efficiently removing both NO and VOCs in a simultaneous process. It addresses the issue of insufficient reduction rates of hydrophobic VOCs by microorganisms, thereby providing a foundation for the optimization and industrial application of flue gas biotechnology in waste-to-energy (WtE) factories.

### 2.4. Photocatalytic Oxidation Technology

As an environmentally friendly advanced catalytic oxidation technology, photocatalytic technology has received widespread attention in removing nitrogen oxide (NOx) and volatile organic compounds (VOCs) from the atmosphere [55]. This technology mainly relies on semiconductor materials such as titanium dioxide (TiO_2_), which generate highly reactive electron hole pairs under light irradiation. These charge carriers can trigger a series of redox reactions, converting NOx and VOCs into harmless carbon dioxide and water [116].

In the photocatalytic removal of NOx, the photocatalytic reaction usually involves the oxidation of NO to NO_2_, which is then converted to nitric acid (HNO_3_) [117]. This process not only reduces NO_X_ emissions, but also brings about the accumulation of HNO_3_ on the surface of photocatalysts, which may inhibit the activity of photocatalysts. To address this issue, researchers have explored adding acid neutralizers (such as CaCO_3_) or designing high-specific-surface-area materials to promote the removal of HNO_3_ [118].

For the photocatalytic oxidation of VOCs, photocatalytic technology can effectively mineralize these harmful organic pollutants into CO_2_ and H_2_O [119]. This process is usually carried out at room temperature, with low energy consumption and no secondary pollution. However, the efficiency of photocatalytic reactions is limited by the availability of light and the performance of photocatalysts. In order to improve the photocatalytic efficiency, researchers have developed various strategies, including doping, composites, and the development of new photocatalysts, to enhance the light absorption capacity and charge separation efficiency [120,121].

E. Luévano Hipólito and Leticia M. Torres Martínez [122] prepared a ZnS/ZnO/CuFeS_2_ thin film based on Earth-abundant elements using natural minerals, thereby demonstrating excellent photocatalytic performance. The catalyst is effective at removing volatile organic compounds (VOCs) such as formaldehyde from indoor air and at generating solar fuels such as hydrogen, methanol, and formic acid from carbon dioxide reduction under simulated visible light. The advantages of this catalyst lie in its natural source, low cost, and environmental friendliness. However, further research is needed to determine its stability in practical applications and feasibility for large-scale production. Gao et al. [123] reported the synthesis and application of TiO_2_ RGO/LDH nanocomposites, which were prepared by the hydrothermal method and exhibited improved photocatalytic degradation of VOCs. The experiment demonstrated that the optimal catalyst loading was 2 wt% RGO/LDHs, which exhibited the highest photocatalytic efficiency in toluene degradation experiments. The advantage of this composite material lies in the introduction of graphene, which expands the photoresponse range and suppresses the recombination of electron–hole pairs. The layered structure of LDHs provides more hydroxide ions, accelerates the oxidation reaction, and generates more free radicals, thereby improving the degradation efficiency of pollutants. However, the disadvantage of this composite material may lie in its complex preparation process and cost control. Bai et al. [124] investigated the synergistic adsorption and photocatalytic process of TiO_2_/SiO_2_ nanocomposites for the removal of volatile organic compounds (VOCs) and fixed them on polyetheretherketone (PEEK) nonwoven filter materials. TiO_2_/SiO_2_ nanocomposites have been observed to exhibit synergistic effects on both adsorption and photocatalytic activity, particularly in the removal of ethyl acetate under solar irradiation. The catalyst’s key advantages lie in its high adsorption capacity and photocatalytic activity, as well as the successful loading of the photocatalyst onto the PEEK nonwoven material through a simple room temperature fixation method, which improves its photostability. However, the proportion of silicon-based materials needs to be precisely controlled to achieve optimal performance, which may be a disadvantage.

The selective removal of specific volatile organic compounds (VOCs) is of paramount importance in photocatalytic processes. In order to enhance the selectivity for particular VOCs, the design and synthesis of photocatalysts with specific adsorption and photocatalytic activities is necessary. As an illustration, in the study conducted by Gao et al. [123] it was observed that TiO_2_-RGO/LDH nanocomposites demonstrated the effective photocatalytic degradation of VOCs, including toluene, methanol, and ethyl acetate. The selectivity may be attributed to the chemical properties and structure of the catalyst surface, which can influence the adsorption capacity of VOC molecules and the separation efficiency of photogenerated charge carriers. Furthermore, the photoresponse range of photocatalysts can be affected by the surface properties of the catalysts.

As an emerging technology, photothermal cocatalysis combines the advantages of photocatalysis and thermocatalysis, and improves the reaction rate and efficiency through the photothermal effect [125]. This technology enhances the thermal activity of the catalyst through the photothermal effect, thereby improving the efficiency of NOx and VOC removal. Research has shown that the photothermal synergistic catalytic technology exhibits the efficient removal of NOx and VOCs in practical applications, and is stable and reliable in multiple repeated tests [55,118].

Photocatalytic oxidation is a process whereby photocatalysts are employed to generate reactive oxygen species (ROS) in the presence of light. These include hydroxyl radicals (·OH), superoxide radicals (·O^2−^), and singlet oxygen. These active species possess considerable oxidation capabilities and are capable of mineralizing pollutants such as volatile organic compounds (VOCs) and nitrogen oxide (NOx) into innocuous small molecules, including carbon dioxide (CO_2_) and water (H_2_O). In the presence of light, the valence band electrons of photocatalysts (such as TiO_2_) are excited to the conduction band, forming electron–hole pairs. Subsequently, the electrons and holes migrate to disparate active sites on the catalyst surface. The reduction of oxygen by electrons gives rise to the formation of superoxide radicals, whereas the reaction of holes with water molecules or hydroxyl groups results in the generation of hydroxyl radicals. The initial stage of the process involves the adsorption of VOC molecules on the surface of the photocatalyst. This is followed by a reaction with the active oxygen species generated on the surface, which results in the oxidation and fragmentation of the VOC molecules. Ultimately, this leads to the conversion of the molecules into CO_2_ and H_2_O. This process comprises a series of stages, including the adsorption, electron transfer, oxidation, and desorption of products. In the case of NOx, photocatalytic oxidation is typically conducted under alkaline conditions, whereby NOx is transformed into NO^3−^ or NO^2−^. In the photocatalytic process, NOx reacts with ·OH to form HNO_3_ or NO_2_, which is subsequently oxidized to form NO^3−^. The photocatalytic oxidation of NOx is typically accompanied by the removal of VOCs, as they frequently coexist in the atmosphere and their reaction pathways are interdependent.

Although photocatalytic technology has shown great potential in the removal of NO_X_ and VOCs, it still faces some challenges, including improving the photothermal conversion efficiency, optimizing catalyst stability and selectivity, and reducing costs. Future research will focus on developing new photothermal catalysts, gaining a deeper understanding of the mechanism of photothermal cocatalysis, and promoting the practical application of the photothermal cocatalysis technology to achieve broader commercial applications.

## 3. Perspectives

Despite the notable advancements in the collective elimination of assorted gas-phase contaminants, the practical deployment of catalysts remains in its nascent stages. To economically and effectively meet rigorous national emission standards, numerous challenges must be addressed. The following areas warrant particular attention in future research.

### 3.1. Catalyst Design

The design of catalysts is of paramount importance for the achievement of synergistic NOx and VOC removal. Researchers have enhanced the catalytic efficiency of catalysts for NOx and VOCs by modifying their compositions and structures. The development of multi-component catalysts, especially those containing transition metals and rare earth metals, has attracted attention due to their excellent redox performance and acidity. In addition, by changing the pore structure of the catalyst, such as introducing mesoporous structures, the diffusion of reactants can be enhanced, and the activity and stability of the catalyst can be improved. The regulation of surface properties, including the modification of acid sites, redox sites, and metal active sites, is also an important strategy for improving catalyst performance.

### 3.2. Reaction Mechanism

A deep understanding of the reaction mechanisms of NOx reduction and VOC oxidation is crucial for catalyst design. Researchers have explored the intermediate generation, reaction pathways, and rate-determining steps in the reaction process through a combination of experimental and theoretical calculations. The coadsorption behaviors of NOx and VOCs on catalyst surfaces and their interactions have significant impacts on improving catalytic efficiency. In addition, real-time monitoring of molecular adsorption and conversion during the reaction process can be achieved through techniques such as in situ infrared spectroscopy (in situ DRIFTS), providing a basis for catalyst optimization.

### 3.3. Deactivation and Regeneration of Catalysts

The deactivation of catalysts is a key issue that needs to be addressed in practical applications. Substances such as sulfur, chlorine, and heavy metals in industrial flue gas can cause catalyst deactivation. Researchers are exploring the antitoxicity mechanism of catalysts and developing effective catalyst regeneration technologies, for example, removing toxins deposited on the surface of the catalyst through heat treatment or chemical washing methods to restore the activity of the catalyst. In addition, by adjusting the composition and structure of the catalyst, its resistance to toxins can be improved.

### 3.4. Practical Application and Engineering

Despite the advancements in NH_3_-SCR achieved through the use of metal catalysts, research into the reduction of multiple pollutants, including NOx and volatile organic compounds, remains limited. In the context of multi-component atmospheric conditions, interactions between reactants are an unavoidable consequence. The influence of volatile organic compounds on the selective catalytic reduction (SCR) reaction is predominantly inhibitory, whereas the impact of the SCR flue gas on the oxidation reaction of volatile organic compounds is primarily facilitative. The removal activity of NO and volatile organic compounds is seldom contingent on a single reactant but rather on the overall atmosphere. It is essential to consider the impacts of other components in the flue gas, particularly SO_2_, water, and heavy metals, on the synergistic degradation process. The influence of these components on the reaction varies depending on the specific reaction involved, such as NO reduction or volatile organic compound oxidation. Therefore, it is crucial to assess the impact of these components on the overall synergistic reaction from a practical perspective, which is the primary objective in NOx elimination and volatile organic compound treatment. The potential applications of catalysts in the treatment of volatile organic compounds (VOCs) and nitrogen oxide (NOx) are extensive. Firstly, the market size of the VOC treatment catalyst industry is projected to exhibit a stable growth trajectory between 2024 and 2030, with a compound annual growth rate of 6% to 8% over the subsequent five-year period. Metal oxide catalysts are employed extensively in the remediation of diverse volatile organic compounds (VOCs) due to their effective degradation of organic contaminants. Secondly, the market for NOx emission control catalysts is also demonstrating a sustained growth trajectory, with considerable potential for further expansion. As environmental policies become increasingly stringent and clean energy technology advances rapidly, this market will continue to demonstrate a stable growth trajectory. Furthermore, the catalyst technology for the simultaneous elimination of VOCs and NOx is regarded as a highly promising multi-pollutant control strategy, with the potential to achieve synergistic reductions in PM2.5 and ozone emissions. The innovation and application of these catalyst technologies will drive market development and enhance market competitiveness.

## 4. Conclusions

The combined catalytic conversion technology of NOx and VOCs has attracted much attention due to its advantages in improving air quality, saving investment costs and space. This technology simultaneously removes these two pollutants through a single catalytic system to achieve more efficient pollution control effects. The main methods include selective catalytic reduction (SCR) technology, photocatalytic oxidation technology, low-temperature plasma catalysis technology, biological purification technology, and the development of new combined catalytic oxidation catalysts. These technologies achieve the conversion of NOx and VOCs through different mechanisms, such as redox reactions, photochemical reactions, plasma excited-state reactions, and biological metabolic processes.

In terms of SCR technology, NH_3_-SCR technology using ammonia as a reducing agent has matured in industrial flue gas denitrification and its application is being explored in VOC catalytic degradation to achieve the synergistic removal of NOx and VOCs. SCR technology has advantages such as the synergistic removal of NOx and VOCs, high catalytic efficiency, commercially mature technology, adjustability, and environmental friendliness. However, it also faces challenges such as high catalyst costs, catalyst poisoning, reaction condition limitations, by-product issues, low VOC oxidation selectivity, increased system complexity, and technical adaptability limitations. Future research directions may include developing more economically stable catalysts and optimizing the reaction conditions to improve system performance and reliability.

In terms of the low-temperature plasma catalysis technology, plasma technology activates gas molecules by generating high-voltage discharge in the gas, thereby removing NOx and VOCs from exhaust gas. Dielectric barrier discharge (DBD), a common non-thermal plasma generation method, generates a large amount of active free radicals and ions by forming a discharge between electrodes, promoting the reaction with pollutants in exhaust gas and achieving harmless treatment. By combining catalysts such as metals or metal oxides, the reaction efficiency and selectivity can be further improved. Pulse electrodynamic dielectric barrier discharge plasma hybrid technology (PDBDH) optimizes the removal efficiency of NOx and VOCs by adjusting the pulse parameters. However, plasma technology still faces challenges in industrial applications, such as high initial equipment costs, maintenance requirements, by-product handling issues, scaling up to industrial scale, potential corrosion issues, ozone generation, and technical operational complexity.

In terms of photocatalytic oxidation technology, as an environmentally friendly advanced catalytic oxidation technology, photocatalytic technology has received widespread attention in removing NOx and VOCs from the atmosphere. This technology mainly relies on semiconductor materials such as titanium dioxide (TiO_2_), which generate highly active electron–hole pairs under light irradiation. These charge carriers can trigger a series of redox reactions, converting NOx and VOCs into harmless carbon dioxide and water. In the photocatalytic removal of NOx, the photocatalytic reaction typically involves the oxidation of NO to NO_2_, which is then converted to nitric acid (HNO_3_). This process not only reduces NOx emissions but also leads to the accumulation of HNO_3_ on the surface of the photocatalyst, which may inhibit the activity of the photocatalyst. In order to address this issue, researchers have explored the possibility of adding acid neutralizers (such as CaCO_3_) or designing high-specific-surface-area materials with the objective of promoting the removal of HNO_3_. With regard to the photocatalytic oxidation of VOCs, it has been demonstrated that photocatalytic technology can effectively mineralize these harmful organic pollutants into CO_2_ and H_2_O. The process is typically conducted at room temperature, with minimal energy consumption and no secondary pollution. However, the efficiency of photocatalytic reactions is limited by the availability of light and the performance of photocatalysts. In order to improve the photocatalytic efficiency, researchers have developed various strategies, including doping, recombination, and the development of new photocatalysts to enhance the light absorption capacity and charge separation efficiency. As an emerging technology, photothermal cocatalysis combines the advantages of photocatalysis and thermocatalysis, improving the reaction rate and efficiency through photothermal effects. This technology enhances the thermal activity of the catalyst through the photothermal effect, thereby improving the efficiency of NOx and VOC removal. Research has shown that photothermal synergistic catalytic technology exhibits the efficient removal of NOx and VOCs in practical applications, and it is stable and reliable in multiple repeated tests.

## Figures and Tables

**Table 1:** Performance of V-based catalysts for the combined conversion of NOx and VOCs.

Catalyst	Method	VOC Type	NOx Type	Space Velocity mL/(g·h)	Optimum Temperature (°C)	Ref.
Ce_10.75_-V-W/Ti-500	impregnation	Toluene, benzene	NO	55,953	420	[70]
Mo_4.5_-V-W/Ti-500	impregnation	Toluene, benzene	NO	55,953	420	[40]
VO(acac)_2_-VTi	extrudate	chlorobenzene	NO	60,000	300	[71]
PrV_5_W/Ti	impregnation	chlorobenzene	NO	50,000	250–450	[72]
V_2_O_5_−WO_3_/TiO_2_	purchase	chlorobenzene	NO	40,000	300	[73]
As/VWT	impregnation	chlorobenzene	NO	40,000	300	[34]
Vox/TiO_2_	purchase	ortho-dichlorobenzene	NO	6000	150–200	[74]
V_2_O_5_/TiO_2_	impregnation	propane	NO	90,000	300	[69]

**Table 2:** Performance of Mn-based catalysts for the combined conversion of NOx and VOCs.

Catalyst	Method	VOC Type	NOx Type	Space Velocity mL/(g·h)	Optimum Temperature (°C)	Ref.
Mn/CeO_2_	impregnation	toluene	NO	10,000	215–300	[89]
Mn/TiO_2_	impregnation	toluene	NO	10,000	239–295	[89]
MnCoOx	impregnation	chlorobenzene	NO	12,000	120	[90]
MnOx nanorods	impregnation	toluene	NO	12,000	275	[91]
MnNb_0.4_Ce_0.2_O_x_	impregnation	chlorobenzene	NO	12,000	250	[92]
Fe-MnO_2_ nanorods	impregnation	toluene	NO	12,000	275	[93]
MnCe/HZSM-5	impregnation	toluene	NO	10,000	300	[94]
V-Mn/Ti	impregnation	toluene	NO	10,000	300	[95]
